# Supplementary material for: Genomic Instability of Circulating Tumor DNA as a Prognostic Marker for Pancreatic Cancer Survival: A Prospective Cohort Study
Source: Cancers (Basel). 2021 Oct 30;13(21):5466. doi: 10.3390/cancers13215466 (PMC8582446; doi:10.3390/cancers13215466)
Supplement: Supplementary file 1 [file cancers-13-05466-s001.zip › Supplementary Figure.pdf]

a.

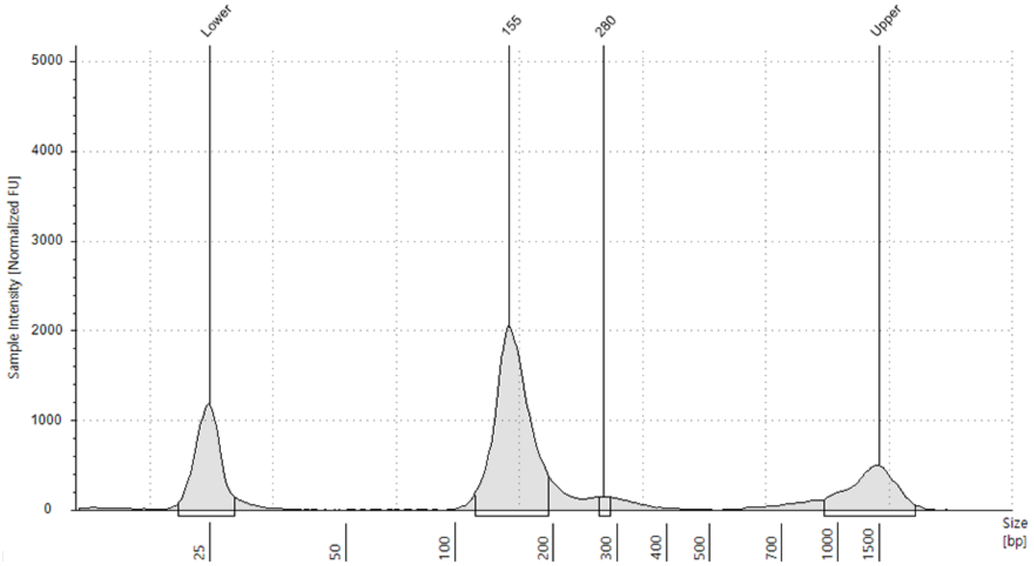

b.

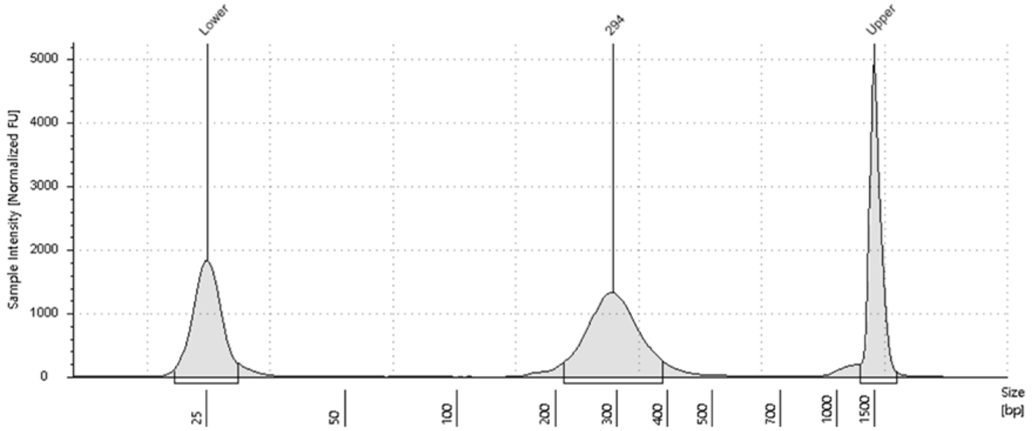

Supplementary Figure S1. Tapestation 4200 profiles of cell-free and tissue genomic DNA. (a) Size distribution of cfDNA gragments. Major peak of cfDNA fragment size was detected between 150 to 180 bp. (b) Sizei distribution of tissue genomic DNA fragments. Major peak of tissue genomic DNA size was detected between 180 to 250 bp.

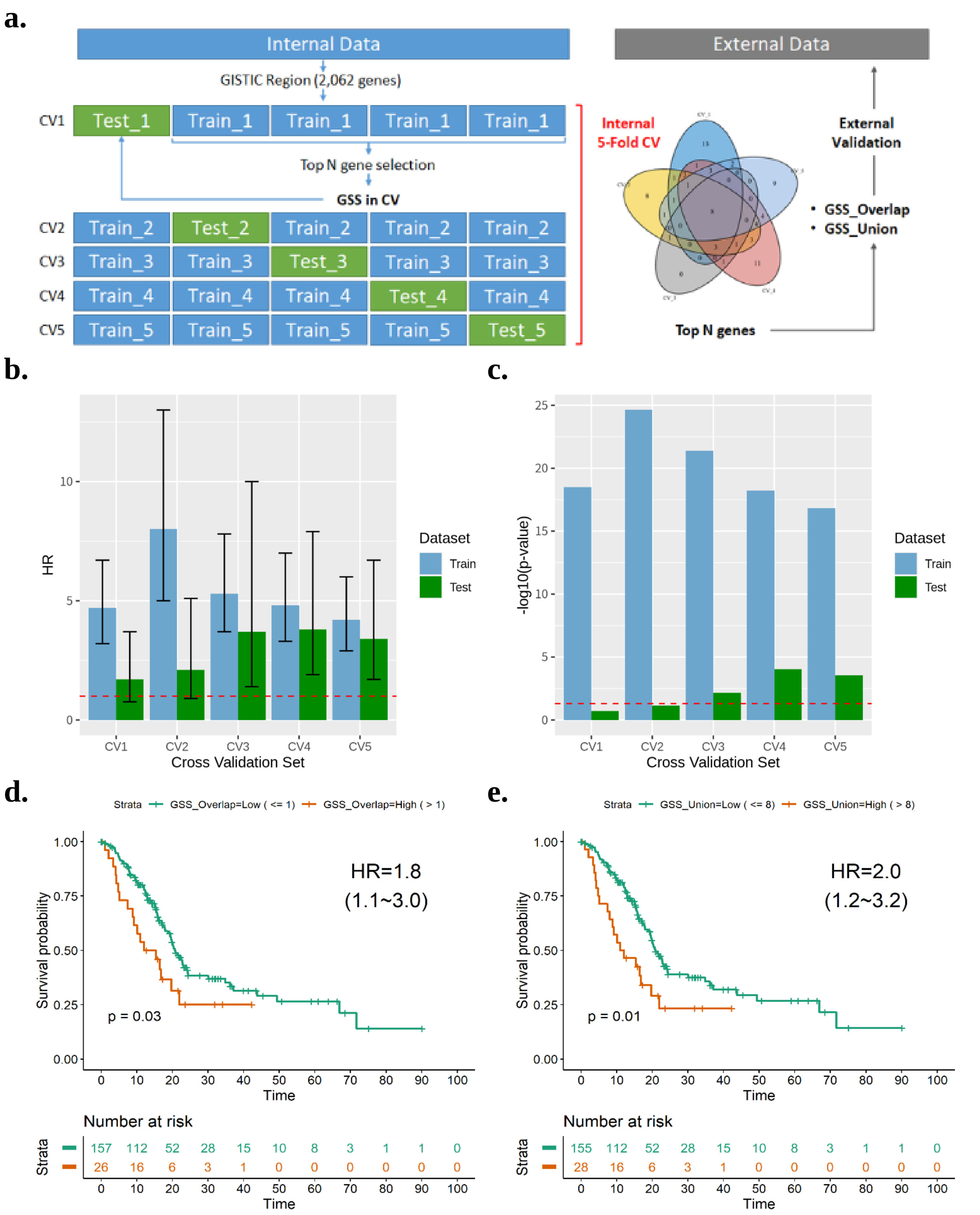

Supplementary Figure S2. Overview of the gene sum score (GSS) analysis and the result of prognostic impact validation. (a) Schematic illustration of the GSS calculation and validation workflow. Five-fold cross validation was carried out for internal validation. Top N significant genes were selected in each of cross-validation sets. GSS_Overlap was calculated using 8 genes repeatedly selected across all five cross-validation sets. GSS_Union was calculated using all 79 genes selected at least once. (b) P-values calculated from overall survival (OS) log-rank test in each cross-validation set. Red dashed line indicates statistical significance cutoff, p=0.05. (c) Hazrd ratio (HR) and 95% confidence interval calculated from univariable cox proportional hazard model. Red dashed lin indicates baseline, HR=1. (d-e) OS curve of GSS_Overlap and GSS_Union in external validation data set.

a.

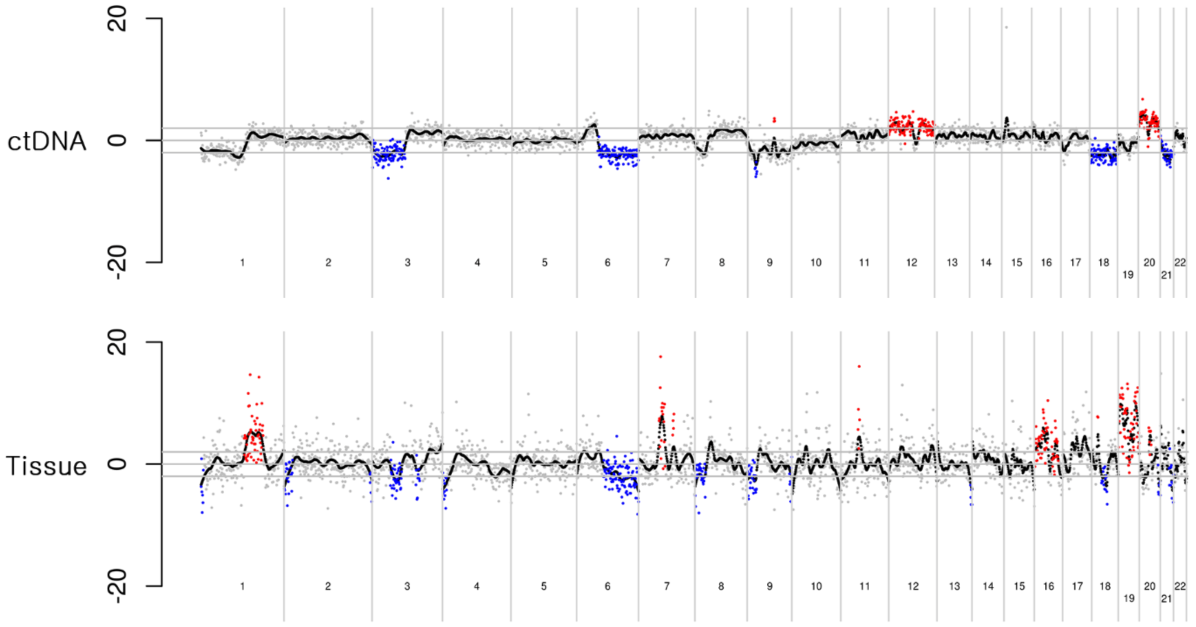

b.

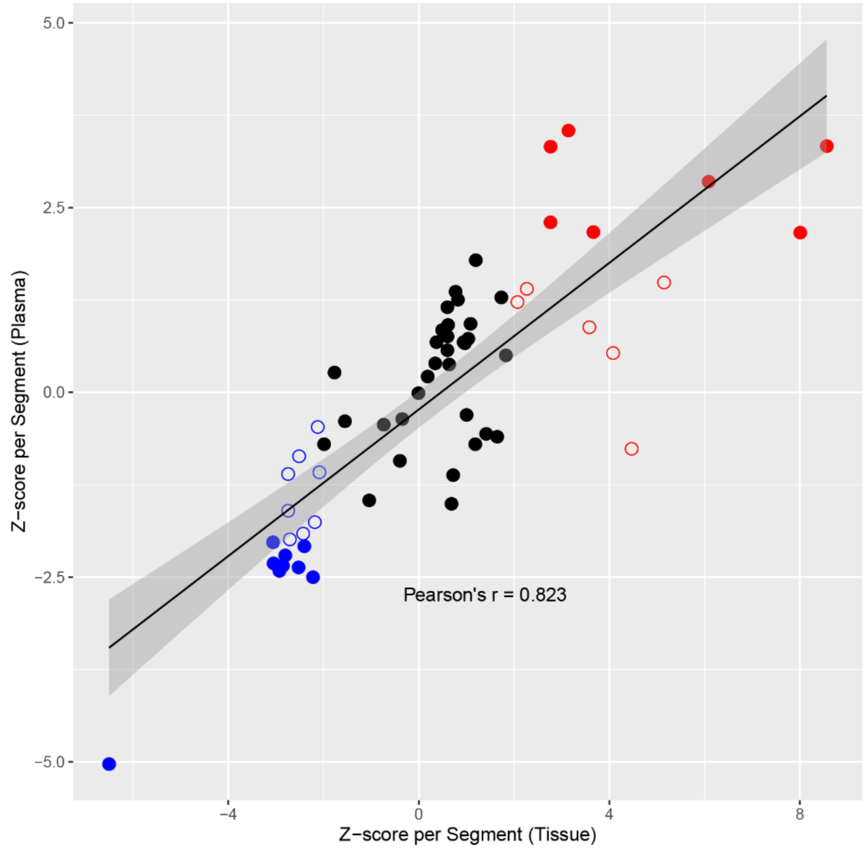

Supplementary Figure S3. Comparison of copy number aberrations (CNA) profiles of circulating tumor DNA (ctDNA) and corresponding tissue genomic DNA. (a) The CNA profiles observed from plasma ctDNA (upper) and matched tumor tissue (lower). Genomic regions identified as deletion (blud) and amplification (red) from segment analysis are highlighted. Each dot corresponds to 1M size bin and Z-score of each bin is indicated on y-axis. (b) Correlations between Z-scores at segment resolution. Segments observed as amplification, neutral, and deletion in tumor tissue were colored in rec, black, and blud respectively. Filled dots indicate segments observed as the same class CNA, amplification or deletion, in both tumor tissue and ctDNA.
